# Exploring communication preferences of trans and gender diverse individuals—A qualitative study

**DOI:** 10.1371/journal.pone.0284959

**Published:** 2023-08-23

**Authors:** Rieka von der Warth, Gloria Metzner, Mirjam Körner, Erik Farin-Glattacker

**Affiliations:** 1 Section of Health Care Research and Rehabilitation Research, Medical Center–University of Freiburg, Faculty of Medicine, University of Freiburg, Freiburg; Germany; 2 Institute of Medical Psychology and Medical Sociology, University of Freiburg, Freiburg, Germany; University of Macerata: Universita degli Studi di Macerata, ITALY

## Abstract

**Background:**

Trans and gender-diverse individuals experience adverse health outcomes that might be due, in addition to other factors, to stigma and discrimination in the health care sector. At the same time, the concept of person-centred care acknowledges the role of patient-physician communication in health care outcomes. This study aims to explore patient-physician communication preferences in trans and gender-diverse individuals.

**Method:**

A qualitative interview study was conducted, including N = 10 participants between February and March 2022. Participants were interviewed using a semi-structured interview guideline, based on previous knowledge in person-centred care and sample specific communication. Participants were asked about their experiences and wishes in patient-physician centeredness. Analyses were conducting using a qualitative content analysis strategy.

**Results:**

Mean age was 29.3 years; n = 6 participants identified themselves within the binary gender concept, while n = 4 identified themselves with a non-binary gender. Communication preferences for patient-physician communication were categorised into four themes: general communication aspects (e.g. active listening); the role of gender during appointments (e.g. appropriate/inappropriate addressing); gender-neutral language (e.g. experiences use of gender neutral language by physicians); own communication style (e.g. early outing and justification). Furthermore, possible contextual factors of patient-physician communication where found (e.g. trusting relationship).

**Conclusion:**

Adding knowledge to communication preferences of trans and gender-diverse individuals, this study was able to identify preferences that are specific to the sample as well as preferences that differ from the cis-gendered population. However, it remains unclear how the patient-physician communication preferences affects health care utilization and outcomes.

**Trial registration:**

German Clinical Trial Register (DRKS00026249).

## Introduction

The gender of trans individuals does not fully and constantly match their sex assigned at birth. The term trans as an umbrella term is widely accepted within the community. It stands for e.g. transgender, transsexual, or genderqueer, but this list could be greatly expanded as individuals identify with a wide variety of terms. Trans individuals might identify with the opposite gender and therefore apply to a binary concept of gender [1]. Additionally, trans individuals might associate themselves with both, between or neither of the genders recognised by society (male/female), often referred to as gender-diverse [1]. Studies suggest, that about 80% of all trans individuals identify with a binary gender concept, whereas 20% identify as gender-diverse such as e.g. 2-spirit, genderfluid, and agender [2].

Overall, trans individuals face a great deal of stigma, discrimination and violence compared to cis-gender individuals [3, 4]. For instance, 54% of all trans individuals in the European Union have reported discriminatory experiences, with 15% experiencing violence within the last 12 months [4]. Stigma and discrimination against trans individuals is also prevalent within the health care system. Physicians are known to have negative attitudes towards this community [5, 6] and with transphobia playing an important role in the provision of health care services [7]. As a result, trans individuals tend to avoid health care services [8, 9], with previous studies suggesting that about 23–39% of all trans individuals fear mistreatment and harassment during consultations [10, 11].

In line with the minority stress theory, this non-acceptance of gender identity in various situations directly leads to worse mental health and overall life satisfaction [12, 13]. In fact, members of the trans community show adverse health outcomes [14] with an increased prevalence of depression and anxiety disorders [13, 15] as well as physical illnesses and suicidality [16–18]. At the same time, trans individuals reportedly have diverse psychosocial needs [19] with gender-diverse individuals being likely to have different experiences and unique needs compared to binary trans individuals [20].

Perceiving the patients unique needs, preferences and values is a core element of person-centred care as highlighted in the integrative model of patient-centeredness [21]. Person-centred care has recently gained importance, including in policy making to promote it on a legislation level and health care system level [22], with one important factor of person-centred care being the physician-patient communication [21]. Clinician-patient-communication reportedly affects several health care outcomes, with better outcomes in, for example, patient-reported outcomes such as quality of life, therapy adherence and emotional health [23]. The concept of person-centered care thereby stresses the individual needs of the patient [24], while still being be tailored to the setting and patient group [25]. In fact, a growing body of research on the cultural tailoring of health communication has been published [26] with a first study describing a culture centred approach to improve physician-patient-communication in trans individuals [27].

However, most studies within the trans community focus on trans specific themes of health care communication [28]. This research mostly focuses on the concept of misgendering in different forms and the correct usage of pronouns [27, 29, 30] and deadnaming [28, 31, 32]. Furthermore, the overall acceptance of the gender is a common topic [29, 33, 34]. For example, genderqueer women described how their looks are being discussed instead of the reason for the appointment [33]. This focus on trans related topics is also reflected by the fact, that instruments assessing TGDI inclusive care focus on the use of pronouns, names and the organisational constructions of physicians´ practices [35, 36].

Only a few studies address non gender-specific aspects of health care communication, with themes such as listening, showing empathy, compassion and shared-decision making [28, 37]. For instance, trans individuals stated valuing an overall respectful communication style [32]. However, it remains unclear which aspects make communication respectful. Furthermore, a systematic review revealed that most research is conducted with a binary gender concept only describing the preferences of binary trans individuals [6].

Overall, personalised care for trans individuals was described as an important step into addressing unmet needs [38], yet many physicians do not have the required knowledge to provide person-centered care to trans individuals [39]. Trans inclusive communication is particularly important in this context, as there is often a close ideological link between gender and body party within the medical field [40], with even subtly trans exclusive language having major negative effects on trans individuals [41]. This experienced language-related stress might even build up over time and have long lasting negative effects on trans individuals [42]. Thus, the aim of this study was to explore preferences for patient-physician communication in trans individuals in the light of person-centred care. Knowledge of this is necessary to adapt physician’s behavior to the sample and provide personalised care for trans individuals.

## Methods

Participant recruitment was conducted by the authors, working at the Medical Center–University of Freiburg. The ethical approval was granted by the Ethics Council of the University of Freiburg (Approval Number: 21–1609) and was registered in the German Clinical Trial Register (DRKS00026249). Results are reported using the Consolidated criteria for reporting qualitative research (COREQ) checklist [43].

### Study design

Recruitment took place between February 2022 and March 2022. An invitation to participate was sent out on various social media platforms (inter alia Twitter and Facebook) as well as to associations of public advocate groups for trans individuals in Germany, with the request to share the invitation with eligible individuals e.g. by means of their newsletter or on their website. For this purpose, the university public relations department created a social media tile with all the necessary information (purpose and content of study, addressed target sample and contact of research group). Based on this, interested individuals were able to contact the research group either by mail or by phone. Detailed study information and a consent form in duplicate was then sent to the interested individuals again either by mail or E-mail. A short socio-demographic questionnaire was also enclosed, with a request to complete it. The questionnaire was later used for the sample description (age, gender, sex assigned at birth; sexual orientation etc.). Data protection measures relevant to the study were recorded in a data protection concept and coordinated with the data protection officers of our institution.

After written informed consent was sent back, individual telephone interviews were conducted with the participants. Participants received a book voucher for 20€ for participating in the study.

It was planned to recruit a total of N = 10 study participants. Considerations regarding the sample size were based on a) feasibility with the available resources and b) whether this was sufficient to achieve data saturation. This consideration is supported by Guest, Bunce [44] and Hennink and Kaiser [45], stating that most themes emerge after approximately six to nineteen interviews. The interviews were digitally recorded and transcribed verbatim by members of the research group. Interviews lasted 33minutes on average (Min-Max: 24-56min). The transcripts were pseudonymised and given a consecutive ID-Number from 1 to 10. As interviews were held in German language, participant´s quotations to illustrate the respective finding were translated into English by the authors of this manuscript.

The interviews were based on a semi structured guideline and therefore contained guiding questions, which, however, still left room for free association. The guideline began with two open narrative questions (Can you tell me about an appointment that you remember as particularly positive/negative in relation to the conversation with a physician?). Based on the free associations of the participants, the interviewer asked further questions regarding their communication with physicians. If participants had difficulties verbalizing their preferences and experiences or had nothing else to say, further questions from the guideline were prompted. Prompting question were based on the following topics: physician’s knowledge on trans/non-binary gender, communication in respect of the received treatments, atmosphere and specific communication behaviours during treatment, and own behaviour during medical treatment. The reported experiences were able to be independent of gender-related appointments.

The guideline was developed based on existing literature on a) experiences of transgender people in medical care [5, 19, 46–50] and b) on known aspects of general health communication and person-centred care [21, 23, 25, 51, 52]. Topic constructs from a questionnaire on communication preferences in chronically ill individuals [53, 54] were also included, as well as the experiences of the researchers in qualitative research design. The guideline can be seen in the S1 File.

### Data analysis

The analysis followed the explanations on qualitative content analysis based on Mayring [55] using a mix of deductive and inductive approach. To meet the quality criterion of intersubjectivity, two researchers evaluated the interviews. In total, the analysis comprised 5 steps. Firstly, RW created a category system top down based on the interview guideline, and applied it to the first 3 interviews, and made some adaptations based on the themes identified bottom up out of the interviews. RW and GM then independently tested the adapted category system on the same three interviews. Afterwards, the category system was adjusted during a joint discussion, again based on the content of the interviews. This process was repeated in steps 3 and 4, with two additional interviews added at each step. In step 5, RW and GM finalised the category system. New main- and subthemes were created in each step, based on the identified themes in the data. RW evaluated all interviews based on the final coding system. This manuscript will only display those categories related to communication preferences. Results will be described in an aggregated way. A full overview of all categories can be seen in the S2 File. The data management was done using MAXQDA 2022 [56].

### Researcher characteristics

RW and GM are both cis-gendered female researchers in the field of health services research and rehabilitation research. They both hold master’s degrees in psychology and have experience with interviewing and qualitative analysis. MK is a professor in psychology and works in the field of in medical psychology and sociology with a focus on person-centred care. EF is a professor in health services research and rehabilitation research with a focus on methodology.

## Results

### Sample

A total of N = 10 individuals participated in this study. Nine individuals indicated that they were assigned the female gender at birth. Five individuals identified themselves as male, with one individual specifying he identifies as trans masculine. One participant identified herself as female. The remaining four individuals identified as non-binary, of which two individuals specified their gender identity as agender and another as non-binary male. Regarding the pronouns chosen, seven persons preferred to be addressed as he/him, one person as she/her. One person preferred to be addressed by name and no pronouns, and another person indicated no preference.

The average age was 29.3 years (min-max: 23–62 years). The sexual orientation of participants included heterosexuality, homosexuality, bisexuality, and pansexuality. One person was currently not sure which sexuality they would like to be classified as and another person did not want to state their sexuality. Further information about the study participants can be found in Table 1.

**Table 1:** Sociodemographic data of participants.

		N	%
Sexual orientation*			
	Heterosexual	2	18.2
	Homosexual	2	18.2
	Bisexual	2	18.2
	Pansexual	3	54.5
	Unsure	1	9.1
	Don´t want to say	1	9.1
Living with at least one partner			
	Yes	6	60.0
	No	4	40.0
Legal family status			
	single	7	77.8
	married	2	22.2
Occupation status			
	Employed without formal training	3	30.0
	Employed with formal training	3	30.0
	In training or in university	4	40.0
At least one parent born abroad			
	No	8	80.0
	Yes	2	20.0
		**Mean**	**Min-Max**
Age (years)		29.3	21–62

* One participant made multiple statements

### Communication preferences in transgender and gender-diverse individuals

We were able to categorise the communications preferences of trans individuals into four main categories: general communication aspects; the role of gender during appointments; gender-neutral language; and own communication style. We further detected possible contextual factors of patient-physician communication, which will be outlined in the following.

#### General communication aspects

First of all, participants talked about some general communication aspects, with content that seemed not particularly related to the specific sample of trans individuals, namely: “taking time”; “active listening”, “shared decision making”; “presence”, “language”;; “patient orientation”; voice”; and “body language”.

Trans individuals wanted the physicians to take time during consultation or be an active listener, e.g. not taking notes while listening, and to be friendly overall, which also related to a friendly voice and an open body language. Furthermore, individuals wanted to be involved into the treatment planning and to be taken seriously by the physician. They also stated it would be nice if the physician was able to re-call the treatment history, as that would reflect an interest in the patient’s health. In addition, a use of language and medical terms adapted to the patient and their pre-knowledge was considered important. Furthermore, participants said, they like it when physicians explain the decisions made, as it helps to understand treatment processes.

*“And mhh, I have been diagnosed with mhh, factor-V-Leiden, the weaker form. Mhh, so a coagulation disorder. And she was not 100% sure, mhh, […] how it affects the hormone therapy. This is why she wanted to get a second opinion from a hematologist and mhh, then she also not only said "Yes, you have to see the hematologist", but also that this is a coagulation disorder, mhh that can have an impact…on the medication, so whether I get the hormones now by gel or by injection, or that the haematologist says "Has no impact on it at all, does not matter". And mhh, she also explained well that you don’t just have to do anything, but why it’s important to do that now.” (Interview 1)*

Additionally, some themes showed content that seemed specific to trans individuals: “acceptance”; “humour”; “private conversations”, and “emotional support”.

The theme of “acceptance” related to the physicians´behaviuor, whether they did not question certain topics, such as being trans or gender-diverse or other topics, such as life-style choices. Participants wanted them to accept the information as the would any other medical relevant information.

“The only doctor where I am gender specific is my endocrinologist and he was given a piece of paper from my therapist where it says "is Trans*" and he accepted that and that was it, because that’s not really his job to question that. There are still some who further question it and somehow want more and…Somehow make a fuss, but the one where I went accepted it and got the piece of paper and continued with it.” (Interview 9)

Furthermore, participants reported that “humour” would often not be appropriate, as they feared negative reactions and discriminatory behaviour. However, “humour” could also be used to help with the overall atmosphere during an appointment and to alleviate the patient´ nervousness.

*Interviewer = “That is, if…just so I understand you correctly, if the doctors are perhaps already relaxed and joking, then you would dare to ask your questions jokingly, because then you do not expect such a negative reaction.”*

*Interviewee = “Exactly, and I wouldn’t have the need to formulate my questions, so somehow perfectly pre-polished in my head, before I can ask them, but I, which just takes time and somehow with such a perceived time pressure then just doesn’t work, but then when the doctors are already somehow relaxed, then I can also ask my questions more loosely. And I can just let the question fall out as it is in my head and…without first thinking about how exactly I have to formulate the question so that it is understandable, but I can just start talking and somehow the question comes through.” (Interview 9)*

“Private conversations” again, were perceived as both positive and negative. Talking about the patient’s private life could be a method to ease the patient, for instance when the physician has been known to them for a long time or maybe already knows of the patient´s family. However, some participants also feared rejection, when talking about the private situation. Some participants also said that doctors should not talk about their own private lives, but should ask patients about their private lives.

*“I’m not there to talk about personal stuff and somehow chat here now, but to get professional help and if I then say something that doesn’t fit into the world view of the person opposite me, I would just wish that a professionalism would be maintained.” (Interview 7)*

The theme of “emotional support” related to the idea that physicians should know about the importance of the treatment for the patient and resonate emotionally in the treatment processes and with experiences of the health care system in general. For example, one participant reported that they saw the surgeon who conducted a transition-related surgery on them years later and that the surgeon seemed to be happy for them being so far in their own transition journey. Another participant reported that they would go to see their family doctor when they had bad experiences with other physicians, as the family doctor would help them deal with the situation.

*“With the endocrinologist and also with the psychologist, that I find…so…let´s say with some topics I find it quite nice, when they ask "And what do you notice then?". Yes, I don´t know, I have suddenly leg hair and then they are happy for me. So, it seems at least. That…that, so little things like that, where…where maybe others are just like, "Huh? Yeah, okay." It´s just, they’re happy for you. They seem to know how important it is to me. So. Um, things like that are nice.” (Interview 3)*

#### The role of gender during appointments

Within the category of “the role of gender during appointments” participants spoke about how often their gender played a role during appointments (themes: “little”/”often”) and if they perceived the addressing of it as “appropriate” or “inappropriate”. Furthermore, the theme of “platitudes and prejudices” was categorised within this category.

Most participants reported that their own gender played only a minor role during their appointments and if it did, it would usually be at a physician where gender normally plays a role to a certain degree, such as endocrinologists, gynaecologists or urologists. One extreme example for “inappropriate addressing” was, when a psychologist talked openly about one participants gender in front of a therapy group, which was perceived as forced outing. Mostly, participants felt discomfort when physicians addressed their gender, especially when the addressing was done without any knowledge of the correct gender-related terms.

*Interviewer = “And how is that for you, when it [the difference between gender and sex in your medical record] is addressed? How does that feel?”*

*Interviewee = “Quite uncomfortable, but actually I am also used to a lot, because in certain areas I was simply not outed for a long time. And accordingly, it was a familiar discomfort.” (Interview 5)*

Examples of appropriate addressing of the gender, according to the participants, were when physicians ask for permission or when they only ask for the overall well-being in respect, for example, the side effects of hormone therapy. Participants also said that an honest interest in their gender and the overall topic would be alright, when addressed in a respectful manner. A respectful manner was defined as e.g. carefully listening and, not asking unnecessary and intimate questions. Yet, participants generally thought addressing the gender should just be done, if the gender related to the reason for the appointment.

*“Because he was also a bit interested in how, for example, this change of name and personal status works and so on, but on a very respectful level, so he didn’t ask me intimate questions or anything, but simply listened to me most of the time and so on, and I really had the feeling that I was being taken seriously.” (Interview 8)*

The theme of “platitudes and prejudices” was used when participants stated that hey feel discriminated against, when physicians use platitudes and outdated terms which are related to prejudices or assumed living choices. Examples were the terms “sex change” or “born in the wrong body”.

*“Yes, or also "You can’t see that at all". That’s always a text where… "Oh, you can’t see that at all, that´s beautiful". Where I then say, yes, that’s very beautiful. "To me you look like a woman". Huh? You know? And that’s the kind of thing that a, I’ll put it in quotation marks, "normal person" would never be addressed like that. That one…a woman, who is by gender, so… .as a woman… "But you don’t look like a woman" or "You look like a woman". No doctor would ask you that.” (Interview 4)*

#### Gender-neutral language

Results on gender-neutral language included the use of the correct name and pronouns by physicians or experience of misgendering, as well as the use of the asterisk to include all genders in written and oral language in German speaking countries. Themes found were: “important”, “not important”, “whishes”, “use by physicians”, and “reactions to mistakes”.

Most participants stated that gender-neutral language was very important to them and that they had even searched on physicians’websites to ascertain, whether gender-neutral language is used before they made an appointment. This would give an idea if the physician itself was using gender-neutral language. One participant said that the importance of using the real name and pronouns would grow with a better passing. This is, as otherwise uncomfortable situations e.g. in the waiting room would occur, as other patients and staff members would be confused if name and appearance do not match their binary gender concept. Two participants further stated gender-neutral language was especially important with physicians who are commonly known for treating just one specific binary sex, such as gynaecologists or endocrinologist.

*“In endocrinology it is important to me, because I have often noticed that they still mostly use the feminine terms and so on, because I don’t know, I think it used to be that mainly female people went there and so on, and that’s why it is simply more pleasant for me if neutral language is used, because then I feel addressed a bit more”. (Interview 8)*

Four participants stated that gender-neutral language was not important to them, three of them identified as binary. However, two of them stated that gender-neutral language was not important to them in oral language as they would be addressed in a binary manner, but thought it was important overall. The non-binary person stated that they are happy if the physicians use gender-neutral language, but do not mind how they are personally addressed. Only one participants stated they did not care about the asterisk or other gender-neutral stylistic elements.

*“So that was important to me before my name… name and personal status change, just that they call me the right name, because you could no longer see it, and I’m just a shy person and it was just uncomfortable for me, if someone asks for Miss [NAME] and I must then stand up and they think why is the guy up now? So that would be me sh… or I found that unpleasant but otherwise so with asterisks or so that’s me personally, I put no value on it, I do not care.” (Interview 6)*

Participants’ wishes mainly included being able to state both, their sex assigned at birth and their gender at the beginning of a treatment. In the best case, physicians should also ask for the patients name as well as their pronouns and favoured use. One participants also said it would be the easiest if physicians just used the surname without using a gender-based salutation. One specific wish, was that participants do not want to be addressed as “trans*women/men”.

“That’s why I still resist being suddenly called a trans*woman. That…I’m a woman!” (Interview 4)

Most participants said that the majority of physicians nowadays respect the wishes of trans individuals and use their correct name and pronouns. Some physicians ask patients how to behave. This and the physicians’ excusal for unintended mistakes was perceived as positive. Participants said that they would then give physicians a second chance if mistakes or misgendering happened by accident.

*“Well, that it is assumed that one fits mhh into this classic binary scheme. Mhh, that’s normal, doctors are no different to the rest of society and somewhere else I’m somehow addressed as a woman. Mhh, so I wouldn’t say that it’s so blatantly negative, mhh, but it’s…well, it’s not good either, it’s not positive either.” (Interview 2)*

Nevertheless, most doctors would initially assume a binary gender system and would not ask about pronouns and gender of their own accord. Furthermore, some participants reported individual physicians just ignore the wishes of trans individuals and still misgender them. If it were be done on purpose, participants would think about going to another physician. Some participants reported om strategies to get physicians to use their correct name or that they had an inner deadline when physician should get it right.

*“And that’s why I kept going there, but I did, the first few times, it actually happened a few times, […], I got up with my head ducked and at some point mhh I was… I just didn’t get up anymore, so basically I just didn’t feel addressed anymore, although I knew they meant me and mhh then she actually went back to her file and looked at something and then she came back and then called me Mr.” (Interview 6)*

#### Own communication style

Participants also reported how they themselves communicate during appointments, e.g. to guide through the conversation, with most of the themes being contrary pairs: “early outing and justification”/”no outing”; “Self-confident behaviour”/”reserved behaviour”; “Defending oneself”/” endure uncomfortable situations”. Furthermore, one theme was found describing the “knowledge about treatment or appointment preparations”.

Some participants felt like they had to self-out themselves early in the treatment, but for different reasons. While some felt it is their duty to tell the physician, especially as it might affect the treatment, others just did not want any questions about their gender. However, the later does not always work according to some participants, as they felt they had to “proselytise” physicians to understand trans individuals. Some participants also reported that they feel not understood to the point where they felt they had to justify the need for any appointment, even when it was not gender related.

*“Sometimes [I] struggle a bit with myself, that I now say okay, I don’t want to lie to a doctor now, mhh or exaggerate something, mhh but find these limits, what is exaggeration and what is just… is presented well enough that they then take you seriously, as I said.” (Interview 10)*

Individuals not outing themselves again had different reasons. Firstly, they did not feel the urge when the appointment was not gender related. However, most of the participants said they did not out themselves because they felt, they were taken more seriously when they just complied with the cis-binary gender system, especially when they are being read as a specific gender, or they just did not feel safe enough to out themselves.

*“Because I have never felt so comfortable in a practice that I thought I would like to address this now. Because, so the dichotomy between–I just take it now that they read and gender me as a woman and it’s all good, um and I don’t have to explain myself or I don’t have to listen to stupid jokes or I’ll make this step and out myself, but that again is exhausting and I don’t know how people will react and up to now I’ve always taken it as a rule to say "Ah, I don’t think I dare to do it now" and…yes, accept that now somehow.” (Interview 7)*

Participants usually reported self-confident behaviour, when they did not understand what physicians said, e.g. when they used too many medicine specific terms or spoke too fast. It was also described when physicians did not explain side effects well enough.

*“Well, I would react differently nowadays I’m like "Ok, mhhh hello? What is that? What is happening to me?” Mhh, as a younger person, I wasn’t so self-confident to ask that and somehow I didn’t demand this right.” (Interview 3)*

On the other hand, participants said their behaviour is often reserved during appointments. Reasons were, that they felt there was not enough time to ask questions or the hierarchy between them. Furthermore, some participants feared to be perceived as stupid when asking questions or felt they could not process the information given to them fast enough to act accordingly during appointments. Sometimes participants would hold back on topics if they did not feel comfortable.

*”If the conversation is going well, if I notice that I’m being listened to and if I feel comfortable, then the nervousness will go away on its own. If I’m not, if it’s not like that and the conversation is somehow stupid or I’m not being taken seriously, then um…I just think very carefully about which topics I’m going to address and which not. And if in doubt, hold back a bit and maybe don’t ask everything I wanted to ask, but only ask half of it and then go back out quickly.” (Interview 7)*

Some participants said that self-confident behaviour is something you can learn, with one participant even stating they had learned to defend themselves against discrimination, when being treated disrespectfully by physicians.

And I think, I’m a human being, I’ve gotten a big…a really, really big back in my life, but when trans* people or uhm people go to the doctor with such things and are treated like that, it means somethings with them. Well, I’m used to that and I think "well, another one", right. Because it might bother me a bit at the moment, but… I recover quickly and then calm down again and then once I got back to normal "What are you doing now? And can you defend yourself?". I’ve learned to defend myself, but for people who…er…they’re completely done with the world, that again you were put in a drawer you don’t want to be in at all or don’t even find yourself in it, right, and they also don´t belong there.” (Interview 4)

In contrast, some participants said they would just endure uncomfortable situations. Some said they are used to it, because they were not yet outed or they feared they would not find another physician so easily. Another reason was that individuals consciously decided when it was worth explaining things and when they would just endure the situation.

*“Well, but I think, I just think in general that society hasn’t got that far yet. And I think I have to think about my own well-being in which situations it’s worth it for me, mhh, when to clarify and mhh somehow take the time and effort. And in which situations it is better for me to live with it.” (Interview 2)*

Lastly, some participants reported that they either have good medical knowledge or that they prepare themselves before medical appointments. This way, they feel like they have a positive impact on the course of the appointment and that physicians take them more seriously. Some participants prepared the appointment due to their nervousness.

*“I usually just try to write down myself somehow, or maybe not necessarily write but dictate in my head what I actually want to say, so that I then sit there and somehow…like before a presentation, I simply don’t know why I’m actually here and don´t know how to say things anymore. Uh, it was also the case from time to time that I wrote myself a cheat sheet, so to speak, why I’m actually here and what the points are that I actually want to address. Because otherwise I’m afraid that I’ll somehow forget or not get it across properly in time.” (Interview 9)*

#### Contextual factors for patient-physician communication

Some contextual factors found seem to be rather general, more than related to the specific sample: “practice team”; “practice design and atmosphere”; “interpersonal fit”; “physician network”; “trusting relationship with physician”. Thus, most participants said that they had at least one physician they have known for a long time and with whom they had a good relationship. Some even had a full network of physician they know would treat them properly. Trusting relationships with physicians helped with feeling well cared for and not hesitating to make appointments when necessary.

However, participants explained that the communication with some physicians just does not work, as one is not on “right wavelength”, meaning there sometimes seems to be an interpersonal mismatch between patient and physician even though the physician behaves respectfully and pleasantly during consultations. In addition, a good patient-physician communication would is just about the physician but also about the team members and how they react to gender nonconforming individuals. Lastly, modern and tidy practice premises are important to feel comfortable in the first place. Having a modern and tidy premises is important as it relates to the perceived competences of the physician. This, in turn is important for a trusting relationship.

*“It´s not just about the doctors, but also about the medical assistants, the employees in the practice. Um, you see that quite often when you look at Google reviews of doctors that people then write “Yes, the doctor is great, but the woman sitting at the reception is super unfriendly and stuff like that”. And I’ve had that experience quite often too…I…um, this gatekeeping and stuff, yes…that’s really important”. (Interview 2)*

One further contextual factor we found was “intersectional experiences of discrimination”. Participants reported that they experiences rejection from physicians due to medical conditions such as an HIV diagnosis or overweight. For instance, one participant reported that a physician assumed they were a sex worker because of them being trans and being HIV positive. Two participants stated they felt like sometimes they were not being taken serious with their problems due to their assigned female sex.

*“I think there are, um, different external things which play role with doctors. Mhh, be it sex, be it weight, mhh… I think these are general prejudices that sometimes exist there. For example, what do I know, I have problems with this and that, "yes, lose weight", where I don’t feel I’ve been treated well, because maybe it’s something else after all. Or I mean, I’m just saying, I’m afraid that something is wrong with me. Yes, you’re just fat!” (Interview 3)*

## Discussion

We conducted a qualitative study to explore communication preferences in trans individuals in the light of person-centred care. We did this with a multidimensional perspective including knowledge on patient-physician communication in other clinical samples and a cultural sample specific perspective on trans individuals. Communications preferences of trans individuals identified in the interviews were classified into four main categories with several sub-thems: general communication aspects; the role of gender during appointments; gender-neutral language; and own communication style. Furthermore, we detected possible contextual factors of patient-physician communication which could affect the communication between physicians and patients. While some of our results are comparable to previous knowledge [e.g. 21, 28], some results seem surprising and specific for the trans sample compared to research investigating communication in other patient samples.

Firstly, some of the preferences found are abstract and frequently reported in previous studies on person-centered care in cis-gendered samples, such as “taking time”. However, previous studies suggest, that the actual time of a consultation is not as important as the atmosphere [23, 51]. Thus, open and trustworthy communication, where patients get the chance to ask all their questions as well as express concerns, needs, and expectations was found to be relevant for several health outcomes [57]. Similarly, we found these aspects within the theme of “patient orientation”, where inter alia being taken seriously by physicians is included. Those topics were found to be among the most relevant patient expectations as regard person-centred care [58] and thus, our findings suggest that trans individuals do not differ in some main preference’s regarding physician communication. Furthermore, one could hypothesis, that the other themes within the set of sample unrelated topics, such as “body language”, “voice” etc. are also related to the overall atmosphere, as is a good clinician-patient-communication described as “broad range of verbal and non-verbal behaviour” within the integrative model of patient centredness [21]. Overall, these aspects are widely comparable to previous research showing trans individuals’ wish for empathy, compassion and shared-decision making [28, 37].

In contrast, some aspects of health communication seem specific to the trans sample compared to that usually described in the literature in other patient samples. “Emotional support”, which is also part of the integrative model for patient-centeredness, is often described as an aspect of the physician behaviour to acknowledge fear and anxiety in patients in regards of illness and the knowledge to react to these [21, 53]. In our study, emotional support was perceived when participants felt physicians related to their progress in transitioning. In this context, emotions were positive, such as being happy or hopeful. One reason for this might, of course, be that trans individuals must be considered a non-clinical sample and thus, health related fear and anxiety might not be the dominant emotions.

Furthermore, talking about the private life and aspects outside of the treatment is usually perceived as positive and described as a holistic view [58, 59]. In contrast, some participants in this study said, they would not like to talk about personal or private topics, as they would fear a judgmental answer or even discrimination. Only participants describing a trusting relationship with their physician as a contextual factor said private conversations could be soothing. Comparable preferences were described for the aspect of humour in this study. Usually, humour is recommended as a desirable communication tool in physicians [60], whereas several of the participants in this study thought it inappropriate.

Most participants reported that their own gender played only a small role during their consultation. In constrast, previous studies described that gender diverse individuals are often asked about their looks and gender [33]. However, participants also said, that their overall acceptance by physicians has increased in recent years, which is in line with the literature [61]. Participants also described how appropriate and respectful addressing could be done, by having a genuine interest in the topic and asking for permission to ask questions or in the context of the treatment. However, physicians should use the correct and current terms and distance themselves from common platitudes. As found in previous research, the usage of correct pronouns and names were important to our sample [27, 29, 30, 32]. Interestingly, some participants, who either identified in a binary gender system or were read as a binary gender, said that gender-neutral language was not important to them in oral language. In contrast, non-binary participants rated gender-neutral language as very important. This finding supports the idea that trans individuals with a binary or non-binary gender might have different needs [20].

Furthermore, the interviewees reported two different communication styles, with one being more self-confident with early outing in the consultation while the other style is described as reserved. The reserved communication style showed overlapping results with previous research, describing that individuals hide their gender identity during consultations in order to be taken seriously [62, 63]. Participants who outed themselves, in contrast often felt they were not taken seriously and found themselves justifying their consultation reason. Houben, Dennert [33] describe similar experience, when genderqueer individuals describe being seen as malingerers. In addition to these two communication styles, most participants described how they prepare for the appointment or how their pre-knowledge affected their health care. Using health information in the interaction with the physician is an important aspect of interactive health literacy [64]. Even though little is known about health literacy in trans individuals, our results indicate that trans individuals might have a good health literacy and show efforts to gather and use health information as described in Hostetter, Call [65]. In contrast, recent results show that trans individuals actually have in fact a health literacy below average comparted to cis-gendered individuals [66]. This might lead to the assumption that the self-assessed health literacy of trans individuals is possibly higher than it actually is.

As regards contextual factors, participants reported a broad range of aspects which might influence their communication with physicians. Participants reported that not just the communication with the physician itself was important, but also with the entire team. It was perceived as possible gatekeeping to care as well as setting the entire emotional context for the visit. Similar experiences were previously described, with the communication with staff members having such an impact that the entire physician consultation afterwards is negatively perceived [67, 68]. Based on our data, we would add, that the atmosphere in the practice itself is also important, e.g. if it is modern, if everything is clean and the state of the equipment and working materials is good. Thus, part of person-centred care is not just the verbal and non-verbal communication patient-physician communication but other factors contributing to it should also be considered. Additionally, most participants in our study described having a trusting relationship with at least one phycician or already had an entire network of phycisians they felt safe with. One recent research suggested, that some trans individuals might value a trusting relationship more than specilised care [68]. Our results suggest, that this might be, inter alia, due to well-known phyisicans possbily helping to regulate emotions after bad experiences.

Lastly, intersectionality is an important framework in public health research [69], even though results are often just descriptive and unable to depict dynamics [70, 71]. Our participants reported multiple discrimination experiences based on sexism, AIDS-stigma, which is intertwined with homophobia and ableism [72], and fat phobia. In accordance with our findings, research showed that transgender people of colour or Asian individuals had different experiences in health care in comparison with white, western and/or cis-gender individuals [73, 74]. Even though our qualitative study cannot describe the complex relations of the perceived oppressions, it does undermine the importance of studying the impact of intersectional discrimination in trans individuals on patient-physician interaction and that health care providers should adapt to intersectional experiences of patients.

### Strength and limitations

This study adds to the knowledge of patient-physician communication in trans individuals when considering person-centred care. To our knowledge, this is the first study exploring communication preferences not only from a culturally adapted and sample specific perspective, but also including overall theoretical knowledge on person-centred care in other clinical samples. However, some limitations should be discussed. For instance, only one participant identified as female, whereas 50% identified as male or transmasculine and 40% identified with a non-binary gender system. This distribution is not represented in the target sample, as most previous research shows higher prevalence of individuals being assigned male but identifying as female [75]. Furthermore, as previously stated only about 20% of individuals identify within a non-binary gender system [2]. Additionally, a possible selection bias can be assumed by the research design with individuals willing to participate in telephone interviews being more competent in communication. Moreover, the predetermined sample size of N = 10 is relatively small and data saturation cannot be fully discussed. We tried to address this problem by adding the interviews successively to the analysis process and indeed the coding system did not change, when adding the last interviews to the analysis. This experience is supported by methodological research [44, 45], with authors recently concluding that a small sample size is in fact sufficient for qualitative research [45]. In addition, the prompting of themes after the opening question might have limited the participant’s answers. Themes were not named within the interview but related open questions were asked, if necessary. Yet, the results show that themes beyond the interview guideline emerged, indicating that the participants talked openly and freely.

Lastly, this research was conducted without a participatory approach [76] and all the data collection, analysis and reporting were conducted by cis-gendered researchers. It is known that in qualitative research the personal characteristics of the research team lead to an unavoidable bias [43] and research on health communication in particular is defined and delimited by researchers and not the community [26]. Thus, results and reporting might be biased by the characteristics of the research team itself and future research should include the target group into the development of the study design and analysis.

## Conclusion

In this study, we explored the preferences of patient-physician communication in trans individuals and our results add knowledge to the field of trans health care within the framework of person-centred care. Overall, our research leads to the assumption that trans individuals show overlapping communication preferences with other patients groups, but differ in some very specific aspects that are related to their history of life experience. With this, we were able to enrichen the knowledge of person-centered care and that the concept should be culturally adapted to target samples.

However, the impact of communication preferences of trans individuals on health care utilization and outcomes remains unclear. Previous research on person-centred care indicates an impact on health outcomes, thus this relationship should be specifically investigated in trans individuals. In fact, good patient-physician interactions are associated with better mental health and less self-harming and suicidal behaviour in trans individuals [77]. However, other literature within trans research addresses further barriers to good health care, such as financial burden [6].

Yet, our results expand the knowledge in culturally adapted communication in physicians and therefore help to optimise trans care. For instance, our results could be used to develop inclusive language recommendations for physicians, which could be integrated into training programmes or clinical communication strategies. Similarly, Townsend and Clark [78] gave first recommendations for a gender inclusive primary care screening with, inter alia, physicians asking openly about questions and concerns patients might have due to their gender. Thus, based on these results a new questionnaire assessing trans individuals’ communication preferences systematically will be developed. Such a questionnaire might be used in practice to adapt communication styles based on the individuals’ need by physicians or with designing communication trainings for physicians.

## Supporting information

S1 File Interview guideline. (PDF)

S2 File Coding system. (PDF)
