# Supplementary material for: Exploring communication preferences of trans and gender diverse individuals—A qualitative study
Source: PLoS One. 2023 Aug 23;18(8):e0284959. doi: 10.1371/journal.pone.0284959 (PMC10446207; doi:10.1371/journal.pone.0284959)
Supplement: S1 File — (PDF) [file pone.0284959.s001.pdf]

EXPLORING COMMUNICATION PREFERENCES OF TRANS AND GENDER DIVERSE INDIVIDUALS– A QUALITATIVE STUDY

**Interview guideline**

## I. Introduction:

− Introducing yourself

− Thank you for participating

- Information about content and procedure of the interview: experiences and communication during medical appointments. There are no wrong answers, please tell completely free.
- Disseration project by Rieka von der Warth; substudy 1 of a total of 3 substudies – the goal is to create recommendations for doctors regarding acting, the first step is about exploring wishes of TGnBG.
- Please talk about medical appointments in general. It doesn’t have to be about your transition or other appointments regarding your gender.
- Duration approximately 30-45 minutes

− Notice the tape recorder/data protection and so on, like in the respondent information!

− Do you have any further questions? Is there anything left to clarify?

***Switch on tape recorder***

## II. Narrative part:

- **Could you tell me about an appointment that you remember positively when it comes to talking to the doctor?**
    - Why was the appointment positive for you?
    - How did you feel during the appointment?
    - What else can you think of?

- **Could you tell me about an appointment that you remember negatively when it comes to talking to the doctor?**
    - Why was the appointment negative for you?
    - How did you feel during the appointment?
    - What else can you think of?

## III. Following questions about specific topics *(if not already addressed in the narrative part):*

---

**Trans*/non-binarity**

---

- **Please describe the role of your gender at a doctor**
    - How do you feel about that? Why?
    - Does this change on whether the treatment is transition related or not?
- **How important is gender neutral language usage of doctors for you?**
    - What impact has gender neutral language on your feelings during medical appointments?
    - How do you feel when doctors aren’t using gender neutral language?
- **Have you had experiences that doctors where familiar about trans* or non-binary topics?**
    - What impact does their knowledge have on the conversation wih doctors?
    - What’s your wishes about the knowledge of doctors?

## Treatment aspects

- **Can you tell about an appointment where you had a positive experience about education about a disease and treatment**
    - Why did you feel positive about the experience?
    - What else can you think of?
- **What's your wishes regarding education about a disease and in conversation with doctors?**
    - What would change for you due to that?

## Atmosphere

- **How should a conversation with a doctor go that you feel to be in good hands?**
    - Which behaviours of doctors play a role for you in this?
    - *For example: being in a good mood, optimism, private sentences now and then, jokes, eye contact, active listening*
- **What is important for you to be able to talk about your feelings freely?**
- **In which cases can you follow a doctor easily?**
    - *For example: short sentences, speaking loud enough, easy language*
- **Could you describe a situation you felt uncomfortable in at an appointment?**
    - Can you remember why you felt uncomfortable?
    - Can you think of anything else?

## Own manner in medical treatment

- **What kind of attitude do you normally have when you go into a conversation with a doctor?**
    - *For example: open/defensive attitude, curiosity, confidence, anxious*
- **Which behaviours could have a positive impact on the conversation?**

## IV. Others

**Is there anything we didn't talk about so far?**

**Do you want to say anything else?**
