# Supplementary material for: Exploring communication preferences of trans and gender diverse individuals—A qualitative study
Source: PLoS One. 2023 Aug 23;18(8):e0284959. doi: 10.1371/journal.pone.0284959 (PMC10446207; doi:10.1371/journal.pone.0284959)
Supplement: S2 File — (PDF) [file pone.0284959.s002.pdf]

Coding System

Exploring communication preferences of trans and gender diverse individuals– a qualitative study

Code	Is being coded, …	Example
**Genreal communication aspects**		
**taking time**	*When people say that doctors (should) take their time in a conversation.*	Mhh, I was once consulted by a doctor, who took his time, mhh where mhh I really was able to ask questions
**active listening**	*When people say that doctors (should) listen actively.*	*What I like about my doctor, is just, that she, always at the beginning, is actually just sitting at the table, you sitting down, have a normal chat and only then she starts typing something on the computer now and then. So she takes a break, mh, so after asking questions she types on the computer and then she might ask another question or something like that.*
**Shared Decision Making**	*When people say if there is enough room for shared decisions and if patients request this.*	Okay…yes, the thing with the wisdom teeth was nothing, which was urgent, but just…mhh…yeah, they left me the choice. If I, uhm…have them extracted or not. I was informed well about every single step.
**precence**	*When people say which general precence doctors (should) have (for example kindness).*	Sure, if Im expecting kind of a service from that person, like it is in that case, mmh… and then somebody is sitting opposite of me with a face like that I'm thinking oh, should I be here or did they want to be by themelself.
**acceptance**	When people say that doctors (should) have an open attitude towards topics they are unfamiliar with. These include for example unbiased rections to specific topics or towards the person or more specifically showing understanding of the topic.<br>The (non-) assessment of topics is also brought up here.	*The only doctor, where I am specifically because of gender is my endocrinologist and he got a sheet from my therapist saying "is trans*" and accepted it and that was it, because it's actually not his job to question this. There are still some who keep questioning and kind of want more and…somehow making a fuss, but the one I went to accepted it and got the sheet and kept going.*

**language**	*If general comments regarding language construction of doctors are made, for example short sentences or scientific vocabulary.*	Mhh, she is using none or not exclusively technical terms anf if she does, she explains them, what's the root of it. And mhh, she doesn't think you're stupid. Well, she is, yeah…I don't know…
**humour**	*When humour in a doctor's appointment is the topic that is talked about.*	I think humour isn't always that appropriate. Like I don't think I'd need more of it.
**private conversations**	*When it's discussed if private side conversations are appropriate.*	So I'm studying Gender Studies and I'm also working in a…mhhhh field of sexualized violence and these are just very politically charged areas and…on one hand I'm happy when people are interested in it but on the other hand it often happened that I'm telling them and then they drop some stupid remark. Or, mhh if my job is even relevant at all, since its kind of thought up problems and I don't know… well…I find it nice, when someone asks about it because I also think that it can have an impact on their work environment or other things, but also I don't like it very much if they depreciate or comment it in some negative way.
**patient orientation**	*When it's discussed, that interview partners want to see themselves being treated as an individual.*<br><br>***WARNING: This code is quite broad and abstract. It is also regarding the desire of participants to be taken seriously, in which cases they are feeling to be taken serious or the feeling that doctors remember their patients and their stories.***	…because I always had the feeling that her interest in patients…actually wasn't very high? Like I think I told her three times what I'm studying and the times she wrote it down in the file again…I thought like…if she's not reading it prior to a discussion.
**voice**	*When people talk about the impact of the voice in a conversation.*	Just the voice, that it's such a smooth voice and that she's taking her time and isn't explaining or saying things super fast or something like that. Yes.

**emotional support**	*When participants tell about doctors, who show up emotions, for example if they're happy for a patient or reactiong to the emotions within the therapy.*	Yeah, I don't know, I'm going there, suddenly I have hair on my legs and they are happy for me. Like, for me it seems like that. That's…that's just little things, where others would be like "So what? Yes okay". That's just…the are happy for you. They seem to know that for me its something of high importance.
**body language**	*When participants talk about in what way body language of doctors is relevant.*	Mhh…and in state of body language it was, I notice that quite often, that mhhh, I had the feeling shes just checking me first. Like mI came into the room and mhh… her gaze was just going top down and back up. And mhh…he…this person always had some…some reading glasses on or…well I don't know if it was reading glasses anymore, anyways, this person always looked over the upper rim of the frames. And it always felt like being eyed, like based on the motto "Ah, are you sure it is?"
**The role of gender during appointments**		
**little**	*When interveiew participants tell that their gender doesn't play a big role at doctors. Those, where gender is always central because of their medical competence (for example gynaecology) are the exception. This category is also coded when doctors address th topic transgender but won't keep talking about it.*	It's actually not adressed very much and doesn't play a bige role in my opinion.
**often**	*When interview participants tell that their gender is a large part of the conversation with doctors, especially when there is no reason.*	I'm under medical treatment at a psychologist for ADHS and I'm going there to get a recipe for the medicine, need to go there like every four months for an appointment of about 5-10 minutes and the penultimate time I outed myself there since I had a new name on my insurance card. And…I could understand that the hormone therapy might be relevant for medicine, I don't know about that, but instead of that he kept asking questions for ageS about the operations and what I actually had and so on.

**inappropriate adressing**	*When participants tell that they consider the addressing of their gender as inappropriate.*	And uhm, and that was the the most awkward, that…was…thart he…it was in the personal meeting, I think there were about three sessions, like single sessions before of the group, and then he kind of outed me in front the group more or less. I didn't find it that bad, but I thought this might be something I would like to do on my own.
**appropriate adressing**	*When participants tell that they consider the addressing of their gender as appropriate.*	The way she did it was absolutely fine, we just chatted a bit, like babbled a bit. She just said, please finish the…the swab thing here and sat there, typed something and said: How is it going at the moment? Do you kind of have this problem? Do you have that problem? Like, or do you feel something? Mhh, well I found that, that was alright, because I mean she is [laughs] [incomprehensible], if I have any medicinal problems or if I am in any other trouble however, that's her job I'd say, to help in any way.
**platitudes and prejudice**	*When participants tell about how doctors use platitudes or predjudices regarding gender and how they feel about it. Examples for phrases and predudice are: "born in the wrong body"; "You can't see it".*	People always say „Yes you are born in the wrong body". And mhh, me for example, I don't think that applies for me, because, mhh, if I was right born as a man, probably I wouldn't have met my partner, since he wasn't even interested in men at the time we met. And mhh, yes, my body doesn't fit completely, just many things don't match, but I can change that. Not on my own, but with help of doctors and that's why I think for example this "in the wrong body", it's not right in my case.

**Gender-neutral language**		
**important**	*When participants tell about if they rate gender-neutral language as important.*	It's quite important for me. Well, since im not in CITY for that long yet, and as I searched for doctors in may I had a look on the websites and for example I also looked if they were gendering on the website.
**not important**	*When participants tell about if they rate gende- neutral language not as important or not important in some situations.*	Mhh by now I don't really care, it will, I think there will be groups who do it and also groups who don't. And I hope that one, the good side wins. One day.
**wishes**	*When participants express wishes about gender-neutral lanuage, for example in doctors offices. This includes for example the question about address or registration of gender in the anamnesis.*	Mhh…so what I would like the very most, when I go to a new doctor's office, I have to fill out an anamnesis anyways, when it would be asked right away "How would you like to be addressed?" "Which pronouns would you like to use?"
**use of doctors**	*When participants tell about how doctors use gender-neutral language. This includes misgender experiences by doctors, not of the team in the office.*	Well, like…I can't remember that this ever happened anywhere, that you would just say your first and last name or only your last name.
**reaction to mistakes**	*…how participants react to mistakes in the usage of gender-neutral language.*	Yes and mhh, also what is also really related to this trans* stuff, I do this thing, that I accommodate most doctors a bit and say "Okay, if you don't call saying Mr. NAME right away next time, that's alright, it might need time." They might have not seen the little note they made on day, it's not so bad, I won't cause big scenes, I'm just saying yes, it's alright. It's fine.

**Own communication style**		
**Early outing and justification**	*…Participants report, that they are proactive in talking about their gender or justify or explain themselves.*	yes and that I...that I have to missionize uhm doctors and also nurses. I find that, always like uhm, remarkable. But that is probably my lot, yes! [laughs].
**Reserved behavior**	*…participants report, that they tend to be cautious/reserved or don't start the conversation themselves.*	Most of the time I tend to let the other person ask questions first instead of telling them things myself, because it's easier to answer them somehow.
**knowledge about treatment or appointment preparations**	*…participants report that they prepare/inform themselves and for example try to control the conversation by asking specific questions so that it's comfortable for them. Can also be coded if there is already prior knowledge without concrete preperation.*	Mh…most times I try to set it up beforehand, maybe not completely but going through it in my head, what I actually say so that I sit there and like…like having a blackout before a presentation and not know why I'm here and not know how nto find the bright words anymore. Uh, sometimes I've written myself a cheat sheet about why I'm actually here and what's the points that I actually want to talk about.
**endure uncomfortable situations**	*...participants report that they are more likely to endure unpleasant situations in order to receive appropriate care.*	Quite uncomfortable, but actually I'm used to it a lot, because I just wasn't outed for a long time in specific areas. And accordingly, it was familiar to be uncomfortable.
**no outing**	*…participants report that they don't adress their gender identity*	No, I…mhhh…am always absolutely compliant, again "absolutely" in big inverted commas…mhh, that I tick my…my assigned one at birth. Mhh…yes because most of the time, at the health insurance or something like that it's also in the file and for reasons that it is in unform, I do it there as well. Mhh, and

		also…maybe a little bit to expain the inconvenience somehow, that's too much for me. Exactly.
**defending oneself**	*…when participants tell about that they fought back after real experience of discrimination. This can happen either through complaint afterwards or by adressing in the conversation.*	I also complanied at the health insurance, of course, that's what I've been doing lately if something like that happens, I do this, that's what people advised me to do and I do it and pass it to the association of public health insurances. And they should take care of it. But I've never heard anything.
**Self-confident behavior**	*…participants speak of being confident while communicating with doctors.*	So, I would react differently to this today, nowadays I'm like " Ok, mhhh hello? What's that? What's happening to me?" Mhh when I was younger I wasn't that confident to ask that and I didn't really claim it right. And that's actually things where I have to say, it actually happened to me at he gynaecologists. Which is really sad.
**Contextual factors**		
**practice team**	*…participants report about the impact of the whole doctors office or the office team on care and well-being.*	No, I just…when I thought about it earlier, uhm, but I told you this in the beginning, that it's not only about the doctors, but also about the assistents and employees in the office. Uhm, what you see quite often, when you have a look at the rewies about doctors on Google, that people say "Yeah, the doctor is great, but the lady at the reception is super unfriendly and so on." And I also felt this quite often, I…uhm..this gatekeeping and so on, that's…that's quite important.
**practice design and atmosphere**	*…participants report about the general design of the medical practice and what impact it has on them. This also includes general organisation of it.*	Yes! Yes, so i think…well I...I'd say a computer from 1970, it won't run the lates ultragame from today and there are reasons. Sure, lot of things develop because they need to. Mhh, I think for this I can compare my dentists well, my old dentist here and my new one. At the new dentist it looks modern, these foils aren't yellowed yet, mhhh that's just something about a bit of cleanliness. It is…it's neat, its clean , there's no dust, that's what I

		expect of a doctor. Well, especially in a treatment room, yeah, I think that's obvious
**interpersonal fit**	*…when participants tell about how they dont match in therapeutic way with some doctors.*	We also weren't on the same wavelength, mhh, like the way he...how he like…mhh, yes, we did…the vibes weren't good, like that.
**physician network**	*…participants report how important it is to have a solid network of doctors, with whom there is a basis of trust.*	But for now I also gathered these doctors around me. Where I really…uh go there relaxed won't be saying "What's coming now again?"…anything, you know? You just go there in a different way.
**Trusting elationship with physician**	*…participants tell about a special relationship with a doctor, which advantages there are and what impact it has on their care.*	I got a, I have a family doctor, who looks after the MVZ PLACE, which is a big uh place for… also people with HIV, for HIV and AIDS, who go there and I know him since I…since I've got the virus in me. That means I know him since 30 years and he's actually a man I've grown old with, that's NAME DOCTOR, he's also in our club, in the board there and does really great things. I really find him great and he also brings me down sometimes when I get out of the hospital again and I say I've been trated like shit again – either as trans*woman or human with HIV or both…nah.
**intersectional experiences of discrimination**	*…participants report about real or hypotheical reasons for further experiences of discrimination in the health care system (for example HIV, obesity etc.)*	I think there are, uhm, different external things, which play a big role for many doctors. Mh, might it be gender, be it weight, mhh…I think those are general pedjudices which exist. Like for example, what I know, I have problems with this and that, "Yeah, lose weight", where I don't feel treated well, because, maybe it's something different. Or I, I think, I am saying I'm afraid of that there's something wrong with me. Yes, you are just fat! To say it in that way.
